# Regulation of Resistance in Vancomycin-Resistant Enterococci: The VanRS Two-Component System

**DOI:** 10.3390/microorganisms9102026

**Published:** 2021-09-25

**Authors:** Alexandra A. Guffey, Patrick J. Loll

**Affiliations:** Department of Biochemistry & Molecular Biology, College of Medicine, Drexel University, Philadelphia, PA 19102, USA; alexguffey9@gmail.com

**Keywords:** vancomycin, antibiotic resistance, two-component system

## Abstract

Vancomycin-resistant enterococci (VRE) are a serious threat to human health, with few treatment options being available. New therapeutics are urgently needed to relieve the health and economic burdens presented by VRE. A potential target for new therapeutics is the VanRS two-component system, which regulates the expression of vancomycin resistance in VRE. VanS is a sensor histidine kinase that detects vancomycin and in turn activates VanR; VanR is a response regulator that, when activated, directs expression of vancomycin-resistance genes. This review of VanRS examines how the expression of vancomycin resistance is regulated, and provides an update on one of the field’s most pressing questions: How does VanS sense vancomycin?

## 1. Introduction

In the early 1950s, the glycopeptide vancomycin was isolated from *Amycolatopsis orientalis* and soon emerged as a promising new treatment for infections caused by penicillin-resistant staphylococci and other Gram-positive bacteria [1,2]. Early studies showed that the compound successfully cleared staphylococcal infections and did not induce resistance in serial-passaging experiments [1,3,4]. Thus, vancomycin was greeted as an attractive alternative to penicillin and was swiftly approved for clinical use by the U.S. Food and Drug Administration in 1958 [1,5,6]. Impurities present in early vancomycin preparations gave rise to significant toxicity, but improved formulations overcame most of these issues; nonetheless, perceptions about toxicity lingered [1,7]. At the same time, alternatives became available (e.g., methicillin), and as a result vancomycin was used only sparingly until the early 1980s, when the increasing prevalence of methicillin-resistant *S. aureus* prompted its use as an antibiotic of last resort [8,9,10,11,12]. Vancomycin also became a popular treatment option for enterococcal infections, which are tolerant of or resistant to some other antibiotic classes [13,14]. This increased use of vancomycin encouraged the development and spread of vancomycin-resistant enterococci (VRE). 

VRE infection was first identified as an emerging clinical problem in the late 1980s, nearly 30 years after vancomycin made its debut [15,16]. Today, VRE are recognized as a pressing clinical concern [17,18,19]. Vancomycin-resistant *E. faecium* is listed among the so-called ESKAPE pathogens (*E. faecium*, *S. aureus*, *K. pneumoniae*, *A. baumannii*, *P. aeruginosa*, and *Enterobacter* spp.) [20], and the World Health Organization has also identified VRE as a high priority for the development of new antibiotics [21]. VRE levels continue to increase, and the prevalence of VRE infections—nearly 55,000 cases reported in the US alone in 2017—emphasizes the need for a deeper understanding of how VRE function [22,23,24]. An important aspect of VRE pathology is the regulatory system that controls expression of the resistance phenotype; this review aims to provide an update on the molecular mechanisms by which vancomycin resistance is regulated in enterococci. 

## 2. Background

### 2.1. Mechanism of Vancomycin Resistance in VRE

**Figure 1:**
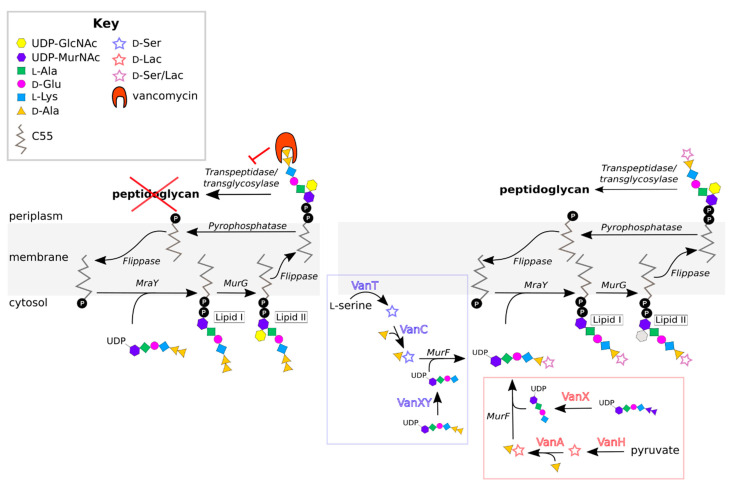
Vancomycin resistance mechanism. Left: In vancomycin-susceptible enterococci, vancomycin binds the d-Ala-d-Ala terminus of the muramyl pentapeptide, inhibiting formation of the properly cross-linked peptidoglycan layer of the cell wall. Right: In VRE, the d-Ala-d-Ala target is remodeled to either d-Ala-d-Ser or d-Ala-d-Lac, neither of which is recognized by vancomycin.

Vancomycin inhibits cell wall synthesis. It does so by binding the d-alanyl-d-alanine (d-Ala-d-Ala) residues of the muramyl pentapeptide portion of lipid II, a precursor in peptidoglycan synthesis (Figure 1) [25,26,27,28]. This binding event interferes with crosslinking of the pentapeptide and formation of mature peptidoglycan (Figure 1) [29], ultimately causing osmotic cell lysis. 

Resistant enterococci have acquired a suite of resistance genes. Several of the associated gene products work together to alter the d-Ala-d-Ala target of vancomycin, preventing vancomycin binding (Figure 1). In different VRE strains, d-Ala-d-Ala is remodeled to either d-alanyl-d-lactate (d-Ala-d-Lac) or d-alanyl-d-serine (d-Ala-d-Ser), thereby reducing the affinity of vancomycin for its ligand [30,31,32,33,34,35,36,37]. This remodeling is accomplished by three essential enzymes encoded in the vancomycin-resistance gene cluster: First, either a pyruvate dehydrogenase (VanH) or a serine/alanine racemase (VanT); second, a ligase that joins d-lactate or d-serine to d-alanine (the naming convention for these ligases is described in Section 2.3); and third, a d-Ala-d-Ala dipeptidase (VanX or VanXY). VanH and VanT convert pyruvate to d-lactate and L-serine to d-serine, respectively [38,39,40], which can then be coupled with d-Ala by the appropriate ligase to form d-Ala-d-Lac or d-Ala-d-Ser [38,39,41,42,43,44,45,46,47,48,49,50,51,52,53]. This dipeptide is added to the UDP-MurNAc-tripeptide by the endogenous MurF enzyme, which has sufficiently broad specificity to accommodate the modified substrate [54]. The resulting (d-Lac/d-Ser)-UDP-MurNAc pentapeptide is incorporated into lipid II and displayed on the exterior of the cell, effectively eliminating the cell’s vulnerability to vancomycin. d-Ala-d-Ala dipeptides produced by the normal cell-wall biosynthetic machinery are cleaved by VanX/VanXY, preventing them from being included in the nascent peptidoglycan chain [55,56,57]. More details about the remodeling aspects of vancomycin resistance can be found in a number of reviews [58,59,60,61,62,63,64,65]. 

### 2.2. Vancomycin Resistance Phenotypes 

VRE isolates are assigned to one of nine types, which are genotypically and phenotypically distinct. These types are denoted by the letters A–E, G, L, M, and N, and their characteristics are summarized in Table 1. Collectively, these types are referred to as the “VRE alphabet” [42,66]. This alphabet should not be considered final, as new resistance types continue to be discovered [67].

Most VRE isolated from human infection sites are *E. faecalis* or *E. faecium*, with the latter being the more prevalent species [80,81]. A-type *E. faecium* are responsible for the majority of nosocomial VRE infections and are particularly difficult to treat due to their resistance to all commonly-used glycopeptide antibiotics [103]. B- and C-type VRE are also clinically significant in humans [81,103,104,105,106]. Due to the differing levels of vancomycin resistance among these types and the various species in which they present, proper typing of isolates is critical for the treatment of VRE infections. This will become particularly important in the event that type-specific treatments are developed. 

The VRE types can be categorized based on whether they use d-Ala-d-Lac or d-Ala-d-Ser to achieve vancomycin resistance. VRE types A, B, D, and M fall into the former group, and types C, E, G, L, and N into the latter. The use of d-Ala-d-Lac versus d-Ala-d-Ser controls the level of resistance observed. d-Ala-d-Lac is bound 1000-times less tightly by vancomycin than d-Ala-d-Ala [26,38,74,107], and thus VRE in which the peptidoglycan precursors contain d-Ala-d-Lac are resistant to high concentrations of vancomycin [45,52,87,108,109,110]. d-Ala-d-Lac is also bound more weakly by teicoplanin, explaining why VRE belonging to types A, D, and M are also teicoplanin-resistant [52,87,108]. 

d-Ala-d-Ser is also bound less tightly by vancomycin and teicoplanin than d-Ala-d-Ala, but the difference in affinity is less dramatic than is seen for d-Ala-d-Lac, with the remodeled precursors being bound ~3- to 8-fold less tightly [37]. Consistent with this modestly reduced binding, VRE types using d-Ala-d-Ser (types C, E, G, L, and N) exhibit only low-to-moderate levels of resistance to vancomycin and teicoplanin [32,34,48,50,51,53,64,93,96,97,98,99,102,111,112].

Expression of vancomycin resistance genes can be inducible or constitutive. VRE exhibiting inducible expression include types A, B, C, E, G, L, and M. For these organisms, precursors containing d-Ala-d-Lac or d-Ala-d-Ser are only incorporated into the cell wall when vancomycin is present; in the absence of the antibiotic, d-Ala-d-Ala is used [32,33,52,58,60,82,83,99,109,113]. In contrast, VRE expressing the resistance genes constitutively (types C, D, and N) produce the alternative dipeptides even in the absence of vancomycin [32,53,58,80,82,83,84,111,112,114]. The mechanisms regulating inducible and constitutive expression of resistance will be discussed in Section 3.

### 2.3. Vancomycin Resistance Genotypes

Historically, the sequence of the d-Ala-d-Lac or d-Ala-d-Ser ligase gene has been used to classify different VRE types [38,39,41,42,43,44,45,46,47,48,50,51,52,53,93]. The nomenclature of these genes parallels that of their respective VRE types: The ligase gene of A-type VRE is referred to as *vanA*, that of B-type *vanB*, and so on. VRE isolates can be typed based on *van* ligase sequence using a variety of PCR techniques [66,100,115,116,117]. Within some VRE types, the sequences of the *van* genes differ sufficiently to warrant subtyping. For example, C-type VRE are subdivided into C1-, C2/3-, and C4-types [44,46,47,80,85]. Other subtyped VRE include B, D, and G [97,118,119,120,121,122,123].

**Figure 2:**
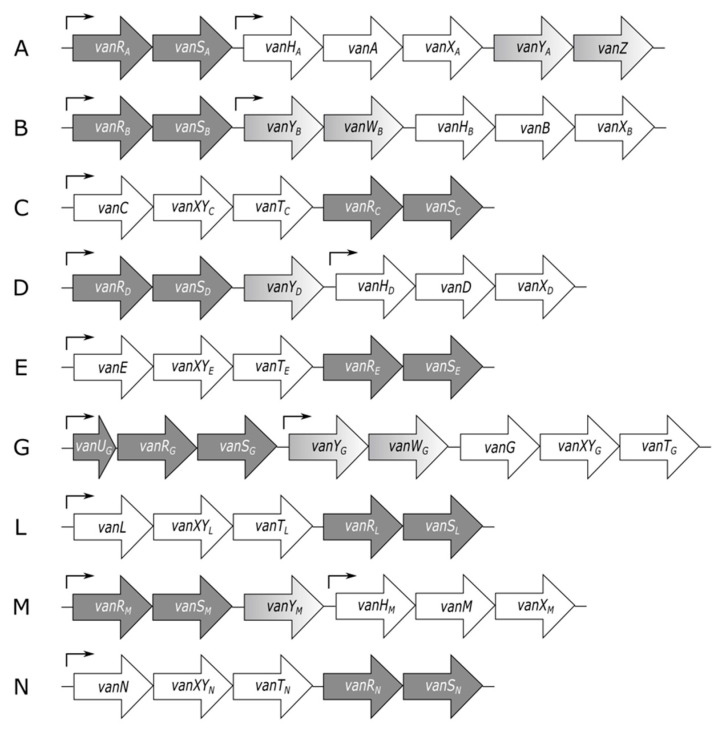
Organization of enterococcal vancomycin-resistance gene clusters for resistance types A–N. Regulatory genes are shown in dark gray, remodeling genes in white, and accessory genes in gray gradient. Arrows indicate the approximate positions of promoters.

Several other genotypic characteristics define the VRE types, including the composition and organization of the resistance-gene cluster (Figure 2). All VRE contain the three essential HAX genes that are required for d-Ala-d-Ala remodeling, as discussed in Section 2.1 [88,124]. In addition to these genes, all operons contain the regulatory genes *vanR* and *vanS* [124], which control how the expression of vancomycin resistance is induced. Additional “accessory” genes are found in some resistance operons (A, B, D, G, M), which may contribute to resistance, but are not essential. A common accessory gene is *vanY*, which encodes a d,d-carboxypeptidase that complements the action of VanX by removing the terminal d-Ala residue from UDP-MurNAc pentapeptides that have escaped remodeling [68,125,126,127,128]. Some VRE lacking the VanY protein still exhibit d,d-carboxypeptidase activity, because their VanXY proteins have dual d,d-dipeptidase and d,d-carboxypeptidase activities [57]. Other accessory genes include *vanZ* in A-type VRE and *vanW* in B- and G-type VRE [129,130]. These genes encode proteins of unknown function, though *vanZ* seems to play a role in teicoplanin resistance [130,131,132]. 

In some VRE, sequences of the regions flanking the resistance operon reveal that the operon was acquired en bloc by transposition. For example, the A-type resistance operon lies within the well-characterized transposon *Tn1546* [15,126,133]. Resistance can also be acquired via conjugation of plasmids harboring the resistance operon [15,43,69,70,71,72,114,134,135,136,137,138,139]. The majority of VRE can transfer resistance genes via conjugation, while types C, D, E, and L-type VRE cannot, suggesting that their resistance genes are chromosomally located [43,49,52,53,75,81,87,93,94,96,99]. 

## 3. Regulation of the Expression of Vancomycin Resistance

Many types of VRE express the vancomycin-resistance phenotype only after exposure to the antibiotic, making the regulation of resistance an intriguing potential target for treatment of VRE. Specifically, compounds that inhibit the expression of resistance could function as antibiotic adjuvants [140], enhancing vancomycin’s potency and restoring antibiotic sensitivity to VRE. Developing such compounds requires a detailed understanding of the regulatory mechanisms governing resistance. This review focuses on these mechanisms; it aims to complement published discussions of this topic, and to provide an update on a key question in the field: How do VRE sense vancomycin?

**Figure 3:**
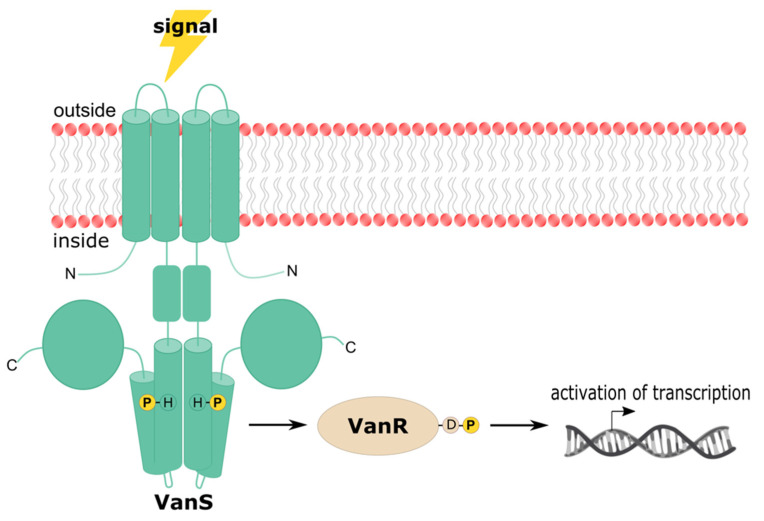
Signal transduction mechanism of the VanRS TCS. VanS receives a vancomycin signal that triggers autophosphorylation of VanS on a conserved histidine residue. VanS transfers the phosphoryl group to VanR, activating VanR. VanR then acts as a transcription factor and mediates expression of resistance genes. In the absence of a vancomycin signal, VanS removes the phosphoryl group from VanR, down-regulating expression of the resistance genes.

Regulation of the resistance phonotype in VRE depends upon the *vanRS* regulatory genes, which encode the VanRS two-component system. A two-component system (TCS) is a type of signaling system found in prokaryotes, archaea, and certain eukaryotes, including plants and fungi. Notably, they are not found in metazoans [141,142,143,144,145]. These systems sense and respond to environmental stimuli via a phosphotransfer signaling cascade [146]. TCSs consist of a sensor histidine kinase (HK) and a cognate response regulator (RR); in the VanRS TCS, these proteins are VanS and VanR, respectively. The signal sensed by the VanRS TCS is vancomycin, and the response is expression of the vancomycin-resistance genes [147,148]. Upon sensing vancomycin, VanS autophosphorylates on a conserved histidine residue (Figure 3). The phosphoryl group is then transferred to VanR [124,149]. When phosphorylated, VanR is activated and upregulates the transcription of the vancomycin-resistance operon [149,150,151]. In the absence of a vancomycin signal, VanS dephosphorylates VanR, switching off the resistance pathway [148,152,153]. This general mechanism appears to be broadly applicable among different VRE types; however, regulatory details vary significantly and are discussed below, beginning with an overview of the architectures and activities of the VanRS proteins.

### 3.1. VanS Architecture and Activity

VanS is a Class-I HK, belonging to the same family as EnvZ [154]. Members of this family are membrane-bound and homodimeric, and contain a periplasmic domain, a transmembrane (TM) domain consisting of two transmembrane helices, a linker region/HAMP domain, a dimerization and histidine phospho-acceptor (DHp) domain, and a catalytic ATP-binding (CA) domain. These domains participate in signal sensing, signal transduction, and/or the catalytic activity of the HK. To date, no structures have been determined for any VRE VanS proteins, and the topologies described herein are therefore inferred from known structures of related HKs and protein prediction software [155,156]. The predicted domains of VanS are listed for each VRE ortholog in Table 2.

#### 3.1.1. Periplasmic Domain 

In the EnvZ family of HKs, the periplasmic domain is thought to detect the activating signal, although in some cases signals may be sensed by other domains (e.g., the TM domain). The VanS periplasmic domain (together with the TM domain and HAMP domain/linker region) lies within the N-terminal half of the protein, which displays considerably more sequence variability than the C-terminal half. The size of the VanS periplasmic domain also differs greatly between the different VRE types, ranging from 12 to 103 residues in length. This heterogeneity in length and composition suggests that periplasmic domains from different VanS orthologs may adopt different structures and thus sense vancomycin differently. Based on the length of the periplasmic domain (Table 2), VanS proteins can be described as either “intramembrane-sensing” or “periplasmic-sensing” HKs. HKs with short periplasmic domains (<50 amino acids) are said to be “intramembrane-sensing,” meaning they detect signals via the TM domain rather than by the periplasmic domain directly [146], and likely sense changes in membrane properties resulting from the signal. All VanS orthologs except VanS_B_ fall into this group. VanS_B_ is categorized as “periplasmic-sensing” HK, because its periplasmic domain contains 103 amino acids. Consistent with this classification, the VanS_B_ periplasmic domain is predicted to adopt a PAS-like structure [157] (P. Rotsides, unpublished results), similar to the ligand-binding periplasmic PAS domains found in multiple other HKs [158,159,160,161,162,163,164]. We caution, however, while sensing mechanisms of different HKs are commonly inferred from the length of the periplasmic domain, this approach alone is not definitive. Possible signal-sensing mechanisms of different VanS orthologs will be discussed in more detail in Section 3.3.

#### 3.1.2. TM Domain

TM domains contribute to signal sensing in some HKs (including possibly VanS). In addition, the TM domain transduces the signal to the catalytic domain, thereby bridging the sensing and catalytic events in the HK [165]. Beginning at the N-terminus, the first transmembrane α-helix (TMH1) passes the membrane from inside to outside the cell; here, it is linked via the periplasmic domain to the second transmembrane α-helix (TMH2), which crosses the membrane again to reenter the cell. 

Molecular structures are key contributors to our knowledge of sensing and signal transduction. However, obtaining structural information for membrane-bound domains like the TM domain is challenging. Nonetheless, a general idea of how signal transduction can function through the TM domain has been developed. This model, based on HK models derived from crystallographic, NMR, and disulfide cross-linking experiments, suggests that signals are transduced by some combination of rotations, translations, and scissoring motions of the TM helices [166,167,168,169]. However, this conceptual framework allows for many possible variations, and in the case of VanS, it is unknown precisely how the TM domain changes conformation in the presence of vancomycin. Indeed, even though different VanRS TCSs share a common signal (vancomycin) and response (resistance gene expression), it cannot be concluded that all VanS proteins share a common signal-transduction mechanism, as underlined by the low sequence identity of the N-terminal regions of VanS orthologs. 

#### 3.1.3. Linker Region/HAMP Domain

All VanS proteins contain a membrane-proximal region connecting TMH2 and the DHp domain, which is responsible for propagating the signal to the DHp and CA domains; deletion of this region abrogates HK activity [170,171]. This linker region contains ~60 amino acids (Table 2). In most VanS proteins, this region is not annotated as containing any specific domain, but in VanS_B_ and VanS_M_ this region is predicted to adopt a HAMP-domain fold [155,172]. HAMP domains are so-named by virtue of their presence in HKs, adenylyl cyclases, methyl-carrier proteins, and phosphatases [172,173]. HAMP-domain sequences are not highly conserved, but they share a canonical two-helix coiled-coil structure [168]; in dimeric HKs, the coiled coils from each protomer associate into a parallel four-helix bundle [174,175,176]. While the linker regions of the other VRE VanS orthologs have not yet been annotated as HAMP domains, they are predicted to be α-helical; thus, given the lack of sequence conservation within HAMP domains, it is entirely possible one or more of these VanS proteins will also prove to contain a HAMP domain. 

#### 3.1.4. DHp Domain

Following the linker region/HAMP domain is the conserved kinase region of the HK, consisting of the DHp and CA domains. These domains are ~70 and ~110 amino acids in length, respectively. The DHp domain earns the first half of its name by contributing to the dimerization of HK protomers; for example, in the EnvZ family of HKs, the DHp domain forms a long helical hairpin, with the two α-helices of each protomer dimerizing to form a four-helix bundle [177,178]. Rearrangements of this helical bundle permit switching between kinase and phosphatase activities. The first third of the first DHp helix harbors the conserved histidine (His164 in A-type VanS), which is autophosphorylated upon HK activation [179,180]. Situated within the aptly-named H box, this histidine residue is absolutely required for signal transduction [165]. The H-box represents the site at which the CA domain docks, bringing the CA domain into close proximity to the histidine phospho-acceptor [165]. In addition to its importance to the autophosphorylation activity of the HK, the H box is required for phosphotransfer and phosphatase activities [181]. Phosphotransfer from the H-box to the RR is made possible by the binding of the RR to the lower portion of the DHp four-helix bundle [141,182]; this portion of the DHp also contains the X region, which is important for phosphatase activity [181]. 

For the A-type VanRS proteins, the aforementioned binding interaction has recently been quantified. Both full-length, detergent-solubilized VanS_A_ and the cytosolic portion of VanS_A_ display low micromolar affinity for VanR_A_, with *K*_D_ values of 1.9 ± 0.7 µM and 6.8 ± 1.4 µM, respectively [183]. Perhaps unsurprisingly, these values fall within the low-micromolar affinity window identified for other HK-RR interactions [184].

#### 3.1.5. CA Domain

In prokaryotic HKs, the CA domain adopts an α/β sandwich topology known as the Bergerat fold [185,186]. Within this domain, the ATP required for autophosphorylation is bound in a crevice within two α-helices, partially covered by a mobile loop called the “ATP lid.” The ATP-binding site and the ATP lid encompass several conserved motifs known as the N, G1, F, and G2 boxes [154,179,180]. Mutations in these conserved motifs can have different effects on HK activity; in particular, some abrogate phosphatase activity, which can cause constitutive expression of the resistance genes. Several such mutations are discussed in Section 3.4. 

### 3.2. VanR Architecture and Activity

VanR belongs to the OmpR family of RRs [187,188] and is divided into two domains: an N-terminal receiver domain and a C-terminal effector domain, joined by a flexible linker [189]. These domains work together to convert the vancomycin signal sensed by VanS into a transcriptional response. There are no published structures of VanR proteins from VRE, but structures are known for many other OmpR-family RRs, including VanR from *S. coelicolor* [190]. These orthologous structures allow us to make structural inferences for the VRE VanR proteins.

#### 3.2.1. Receiver Domain 

The receiver domain accepts the phosphoryl group from VanS, with phosphorylation occurring on a conserved aspartate residue (Asp53 for VanR_A_). In OmpR-related RRs, this aspartate is situated at the end of the third β-strand of an α/β sandwich [189]. Once phosphorylated, the receiver domain undergoes a conformational change, allowing it to dimerize at a conserved α4-β5-α5 interface [191].

#### 3.2.2. Effector Domain 

The effector domain of VanR is a winged-helix DNA-binding domain [187,192], with helix α8 serving as the recognition helix of the winged-helix motif. Insertion of this helix into the major groove of the DNA allows VanR to bind to its target promoters, thereby facilitating expression of the resistance genes, as well as upregulation of the *vanRS* genes. VanR targets either one or two promoters, depending on the relative orientations of the *vanRS* and *vanHAX* genes. For resistance operons in which the *vanRS* genes are located upstream of the remodeling-enzyme genes (types A, B, D, G, and M; see Figure 2), VanR recognizes two distinct promoters, one controlling expression of *vanHAX* and the other controlling expression of *vanRS* [150,151,193,194]. However, for operons in which the *vanRS* genes lie downstream of the remodeling genes (types C, E, L, and N), only a single promoter is used [53,83,94,193]. VanR_A_ and VanR_B_ have been shown to bind their DNA targets in both the phosphorylated and unphosphorylated states; however, transcription of resistance genes is achieved only when VanR is phosphorylated [129,148,149,150,152,153]. A plausible model to explain this observation is that phosphorylation-induced dimerization enhances DNA binding, either by conformational changes that give rise to optimal orientation of the effector domains and/or through an avidity effect [150,151,195,196]. This process is then reversed by dephosphorylation [197,198,199,200,201].

The activities of the VRE VanR proteins will be more clearly understood once structures are determined for these proteins. Although it is possible to formulate models of VanR architecture by examining structures of related RRs, the specific details revealed by true experimental structures may provide insights into treating VRE. For example, therapeutics that disrupt the VanR-VanS or VanR-DNA interactions might restore vancomycin sensitivity to VRE, and are thus worth investigating. 

### 3.3. VanS Sensing of Vancomycin 

The expression of vancomycin resistance is initiated when VanS detects vancomycin in the periplasmic space. The mechanism by which this occurs remains one of the principal open questions in the field. Addressing this question has proven challenging, at least in part because VanS is an integral membrane protein, and therefore a difficult subject for biochemical and biophysical analysis. Furthermore, different VanS orthologs may employ different vancomycin-detection mechanisms, meaning that insights gleaned from one system cannot necessarily be translated to another. 

**Figure 4:**
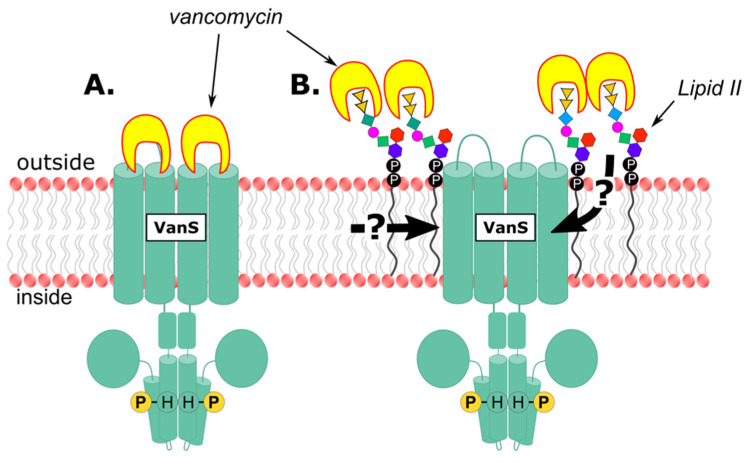
Hypothetical models illustrating vancomycin sensing hypotheses. (**A**) Direct sensing by binding of vancomycin to the periplasmic domain of VanS. (**B**) Indirect sensing of vancomycin by detection of some not-yet-determined, downstream effect resulting from the vancomycin-d-Ala-d-Ala binding event. Two hypothetical sensing routes (detecting membrane stress or Lipid II buildup) are indicated by the heavy black arrows.

Broadly speaking, VanS could detect vancomycin via two distinct mechanisms: It might detect the antibiotic directly, by binding to it (Figure 4A), or indirectly, by sensing some downstream effect of vancomycin activity (Figure 4B). There is currently little consensus as to which model is correct for any given VRE type. 

Direct binding provides the most conceptually straightforward model. A direct-binding mechanism has been most convincingly demonstrated for the non-VRE species *Streptomyces coelicolor*, which expresses the VanS ortholog VanS_Sc_. A direct interaction between vancomycin-VanS_Sc_ was deduced using a vancomycin photoaffinity probe, which was shown to label native protein in *S. coelicolor* membranes, as well as recombinant VanS_Sc_ in *E. coli* membranes [202]. Unlabeled vancomycin effectively competed with the vancomycin photoprobe, arguing for the specificity of this interaction. While this result is compelling, it must be noted that these studies employed membrane preparations that contained lipid II; vancomycin binding to lipid II would tend to produce a high local concentration of the antibiotic, which could give rise to labeling from nonspecific proximity. However, this concern is lessened by a recent NMR study that shows a direct interaction between vancomycin and a peptide corresponding to the periplasmic domain of VanS_Sc_ [203]. 

In contrast to the direct-binding model, indirect-detection models include any mechanisms that do not involve a direct physical interaction between vancomycin and VanS. One such indirect model suggests that VanS senses increased lipid II levels resulting from vancomycin’s inhibition of transglycosylase and transpeptidase enzymes (illustrated in Figure 4B) [204]. An alternative model posits that VanS senses changes in membrane properties resulting from vancomycin activity. A precedent for the latter model may be found in other HKs that are thought to alter conformation within the membrane in response to changes in temperature, thereby functioning as “molecular thermometers,” such as DesK in *Bacillus subtilis* or CorS in *Pseudomonas syringae* [205,206]. 

Yet another potential indirect-detection mechanism involves the regulation of VanS by one or more additional proteins. Many HKs are known to be regulated by other proteins, which may be upregulated in the presence of the HK stimulus, or may themselves bind the stimulus [207,208]. For example, the stress-sensing HK LiaS of *B. subtilis* is regulated by the small membrane protein LiaF. LiaF inhibits LiaS, turning “off” expression of the LiaS-regulated genes in the absence of signal [209]. LiaS adopts the same domain architecture as most VanS orthologs, having two transmembrane helices and a small periplasmic domain [210]. Hence, the LiaS example suggests that regulation by auxiliary proteins is at least formally possible for VanS proteins; however, to our knowledge this mechanism has not been carefully investigated for any VanS orthologs. 

#### 3.3.1. VanS_A_ Sensing of Vancomycin

VanS_A_ is the most well-studied of the VRE VanS orthologs, and much evidence is available that relates to its mechanism of vancomycin sensing. Early work focused on determining which compounds activate VanS_A_, with activation being assessed by the ratio of d-Ala-d-Lac- to d-Ala-d-Ala- in peptidoglycan precursors, the activity of the d,d-dipeptidase VanX, or the expression levels of the *vanHAX* genes [29,211,212,213,214]. These experiments revealed that VanS_A_ is activated by a myriad of antimicrobial agents that interfere with cell-wall synthesis and/or compromise the integrity of the cell envelope. These compounds include glycopeptide antibiotics such as teicoplanin, avoparcin, ristocetin, and of course vancomycin, as well as structurally unrelated compounds such as bacitracin, amphomycin, moenomycin, penicillin G, and tunicamycin. The structural heterogeneity of these different activators would seem to argue against a direct-binding model, since it is unlikely that a single binding site could recognize such a diverse array of ligands. Because most of the activating compounds listed interfere with cell-wall biosynthesis, a model in which VanS_A_ senses vancomycin by detecting lipid II accumulation appears viable [29,212]; however, such indirect sensing mechanisms have not been thoroughly investigated for VanS_A_. We note that not all activating compounds need act by the same mechanism. For example, glycopeptide antibiotics appear to be more potent activators of VanS_A_ than other agents [212,213,214]; hence, it is possible that VanS_A_ directly binds vancomycin and other glycopeptides, whereas other compounds activate the enzyme through different means.

Late-stage intermediates in cell-wall biosynthesis, such as lipid II, are not the only potential candidates for activating VanS_A_. This was shown by Ulijasz et al., who devised a VanS_A_ reporter system in *B. subtilis*, using the *PvanH* promoter fused to a *lacZ* gene [147]. They found that fosfomycin and d-cycloserine (albeit at high concentrations) could activate their reporter, as well as cell-wall hydrolytic enzymes such as lysozyme and mutanolysin. These treatments cause the build-up of a wide range of different peptidoglycan precursors and breakdown products. The structural heterogeneity of these molecules again makes it unlikely that a single binding site in VanS_A_ directly recognizes them. However, membrane stress is a common consequence of all of these treatments, and may therefore be a more credible candidate for the activating signal. Consistent with this idea, the membrane-perturbing agent chlorhexidine gluconate also activates VanS_A_, as revealed by RNA-seq analysis in *E. faecium* [215]. Control experiments in a Δ*vanRS* strain showed no increase in *vanHAX* transcript abundance, implicating VanS in sensing the chlorhexidine [215]. 

In addition to the cellular assays for VanS_A_ activation described above, activation can also be probed in the purified enzyme, by measuring its autophosphorylation, phosphotransfer, and dephosphorylation activities. If vancomycin directly activates VanS_A_, it should increase autophosphorylation and phosphotransfer activity, decrease phosphatase activity, or both. However, detergent-solubilized VanS_A_ displays no change in any of its activities in the presence of vancomycin [183]. Adverse effects of detergent micelles on VanS_A_ activity can be ruled out, since when VanS_A_ is reconstituted in either amphipols or nanodiscs, its autophosphorylation and dephosphorylation activities also do not change in the presence of vancomycin [183,201]. These in vitro findings argue against a direct-binding model for VanS_A_. 

Despite this large body of evidence favoring an indirect-detection model for VanS_A_, evidence also exists supporting a direct-binding model [216,217,218,219,220,221]. A sedimentation-velocity experiment performed using detergent-solubilized VanS_A_ revealed a shift in the sedimentation coefficient of VanS_A_ in the presence of vancomycin, suggesting that vancomycin induces a conformational change in the protein, presumably via a direct interaction [222,223]. Additionally, vancomycin was found to alter the circular dichroism spectrum of detergent-solubilized VanS_A_, which has been interpreted as evidence for direct binding of the antibiotic, with a dissociation constant *K*_D_ of approximately 70 μM [222,224]. Interestingly, this relatively high *K*_D_ value is roughly one to two orders of magnitude higher than the antibiotic concentrations required to inhibit growth of antibiotic-sensitive Enterococci [216,217,218,219,220,221], raising questions about whether this binding is relevant to activation of the resistance phenotype. An additional caveat is that these results were obtained in the presence of detergents, which can alter the conformations and activities of many membrane proteins. In particular, VanS_A_’s autophosphorylation activity is highly sensitive to detergents [183,225].

Finally, as we weigh indirect vs. direct sensing mechanisms for VanS_A_, we note that models can be conceived that combine elements of both mechanisms. For example, VanS_A_ activation might entail recognizing a vancomycin-lipid II complex, rather than the antibiotic alone. Support for this idea comes from the *S. coelicolor* system, where VanS activation only occurs when vancomycin binds its d-Ala-Ala target [226], even though vancomycin has been shown to bind directly to the sensor’s periplasmic domain [203].

In summary, while the preponderance of evidence currently points toward an indirect-detection mechanism for VanS_A_, tantalizing data also exist that support a direct-binding model. Ultimately, this question will not be resolved without further study.

#### 3.3.2. VanS_B_ Sensing of Vancomycin

The vancomycin-sensing mechanism of VanS_B_ is less well-studied than that of VanS_A_, but the cumulative weight of the evidence points to a direct-sensing mechanism. First, VanS_B_ is activated only by vancomycin [212], in stark contrast with VanS_A_. In the preceding section, we noted that it is difficult to conceive of how VanS_A_’s small periplasmic domain would be able to recognize the structurally diverse set of molecules that activate resistance, providing suggestive support for an indirect-binding mechanism for VanS_A_. Conversely, VanS_B_’s narrow specificity for its activator makes it plausible that the protein does bind vancomycin directly.

VanS_B_’s ligand preference maps to its periplasmic domain, with mutations in this region altering ligand specificity and rendering B-type *E. faecalis* resistant to teicoplanin [227,228]. These mutations will be discussed in more detail in Section 3.4. The VanS_B_ periplasmic domain does not exhibit a high degree of sequence homology to any domains of known structure; however, threading experiments predict that it can adopt a PAS-domain fold (P. Rotsides, unpublished results). This would be consistent with the lack of homology with other proteins, since PAS domains typically exhibit low pairwise similarities with one another, and contain no highly conserved residues [157]. A PAS domain in VanS_B_ would not be unprecedented among HKs, as a number of other sensor kinases possess periplasmic PAS domains, including CitA, PhoQ, and DcuS [163,164,229]. However, VanS_B_ is the only enterococcal VanS ortholog for which a periplasmic ligand-binding domain is predicted. Combined with the specificity of VanS_B_ for vancomycin, this observation suggests that VanS_B_ may sense vancomycin by direct binding, which could make it an outlier among the VRE VanS proteins.

### 3.4. Inducibility of Vancomycin Resistance Expression

In the canonical model for VanRS function, exposure to vancomycin leads to expression of the vancomycin-resistance genes, via the intermediate steps of activation and autophosphorylation of VanS and subsequent phosphotransfer to VanR. However, in practice, vancomycin induces expression of resistance in only a subset of VRE types: A, B, E, G, L, M, and some C. In contrast, in other VRE types (D, N, and some C) vancomycin-resistance genes are expressed constitutively, regardless of whether the antibiotic is present. By comparing and contrasting the inducible and constitutive systems, we can gain insights into mechanisms of VanRS signaling.

As noted earlier, VanS possesses both kinase activity (i.e., autophosphorylation and phosphotransfer to VanR) and phosphatase activity (dephosphorylation of VanR). In inducible systems, vancomycin induces expression of resistance by tipping the kinase/phosphatase balance in favor of the former [148]. However, in the non-inducible VRE types (C, D, and N), VanR is constitutively phosphorylated. This might result from VanS proteins having constitutively active kinase or defective phosphatase activities, or from the complete loss of VanS (note that in the absence of VanS, VanR can still be phosphorylated by small-molecule phosphoryl donors such as acetyl phosphate). Since phosphorylated VanR has a long half-life (up to 17.6 h), resistance genes can be transcribed for a considerable amount of time following a phosphorylation event [149].

**Figure 5:**
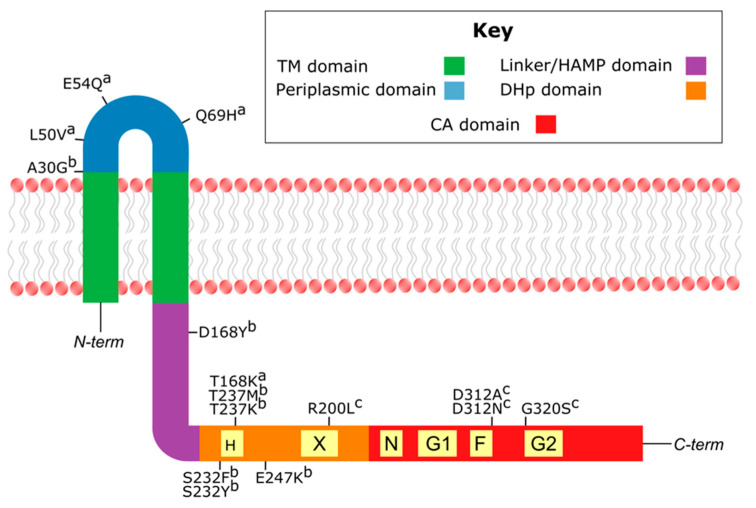
Domain architecture of VanS showing notable mutations affecting inducibility. Domain identities are found in the key. Conserved motifs (H, X, N, G1, G, and G2 boxes) are in yellow. Mutations are identified based on amino acid numbering of their respective VanS ortholog, designated by superscripts (^a^ corresponds to VanS_A_, ^b^ to VanS_B_, and ^c^ to VanS_C1_).

Much of our knowledge about the inducibility of vancomycin resistance is derived from analysis of mutant VanS proteins with constitutive kinase activities/loss of phosphatase activity [64,193]. A handful of these mutants are shown in Figure 5 and will be discussed in the following sections.

#### 3.4.1. Mutations Abrogating Inducibility of Resistance

Mutations affecting inducibility of vancomycin resistance were first identified in B-type VRE grown under teicoplanin selection [227,228,230]. Amino-acid substitutions leading to the constitutive expression of vancomycin resistance were found in the DHp domain, both in the H box (S232F, S232Y, T237K, and T237M) and immediately downstream of the H box (E247K) [227,228,230]. These mutants are also resistant to teicoplanin, as constant remodeling of peptidoglycan precursors eliminates the d-Ala-d-Ala target of teicoplanin. To our knowledge, the enzymatic consequences of these mutations have not been experimentally tested, but clues about their effects can be found in other HKs, in which similar substitutions within and near the H box abrogate phosphatase activity [231,232]. Hence, it appears likely that loss of phosphatase activity explains the constitutive expression of resistance associated with substitutions in the VanS_B_ H box. Supporting this notion, a recent mutational study of VanS_A_ showed that substitution of residue T168 (corresponding to residue T237 of VanS_B_) decreased VanS_A_ phosphatase activity without affecting autophosphorylation activity [183].

Loss of all VanS activity should also lead to a constitutively resistant phenotype, as VanR can still be activated by endogenous small-molecule phosphoryl donors such as acetyl phosphate. Consistent with this idea, when a stop codon is inserted after codon 30 in VanS_B_, constitutive resistance to both vancomycin and teicoplanin results [233].

For C-type VRE, some isolates (typically C1) express resistance constitutively, while others (C2/3 and C4) exhibit inducible resistance. The constitutive phenotype appears to map to substitutions in the DHp and CA domains. Comparison of VanS sequences from constitutive and inducible strains revealed several notable substitutions associated with constitutive behavior: R200L, D312N, D312A, and G320S [83]. Residue 200 is found in the X region, and mutations to the corresponding region of the DHp domain in EnvZ have been shown to disrupt phosphatase activity [181]. Hence, R200L appears to provide another example in which loss of VanS phosphorylation activity causes loss of inducibility. 

The VanS_C_ substitutions D312N, D312A, and G320S fall between the F and G2 boxes of the CA domain. In the related Class-I HK EnvZ, mutations to the F box primarily affect phosphotransfer, while mutations to the G2 box affect all three enzymatic activities [181]. Thus, the mechanistic basis of these mutations is not yet clear. However, in a B-type clinical isolate of VRE, a six-residue deletion in the G2 box significantly disrupted only the phosphatase activity of VanS_B_ [234]. Tentatively, then, we suggest that the D312N, D312A, and G320S mutants abrogate inducibility by decreasing VanS_C_ phosphatase activity.

#### 3.4.2. Mutations Affecting Resistance to Teicoplanin

Certain point mutations within the sensor region cause VanS_B_ to be activated by teicoplanin. For example, an *E. faecalis* strain selected for growth in the presence of teicoplanin was found to contain a A30G mutation in its VanS_B_ protein, which conferred teicoplanin resistance by making the resistance genes inducible by teicoplanin [227]. Residue 30 is predicted to lie at the beginning of VanS_B_’s periplasmic domain, suggesting that the A30G mutation may alter glycopeptide recognition by the periplasmic domain. Alternatively, it is possible that wild-type VanS_B_ can bind to teicoplanin, but is unable to transduce this detection event to the protein’s catalytic region. If this is true, the A30G mutation, lying as it does at the junction between the protein’s first transmembrane helix and its periplasmic domain, may enhance the efficiency of signal transduction. Consistent with this notion, other teicoplanin-resistant VanS_B_ mutations have been found either in the HAMP domain (D168Y) or between the HAMP and DHp domains (E221G) [227,233]. Because this region is important for signal transduction, the ability of these mutants to confer teicoplanin resistance might also reflect more efficient signal transduction in the presence of teicoplanin.

The response of A-type VRE to teicoplanin can also be altered by substitutions in the sensor region of VanS_A_, including L50V, E54Q, and Q69H, all of which fall within the predicted periplasmic domain [235]. In these variants, transcription of resistance genes cannot be induced by teicoplanin; however, they retain their inducibility by vancomycin. This is consistent with a direct-binding model in which VanS_A_ recognizes glycopeptides via its periplasmic domain, with the ability to sense teicoplanin being specifically lost in the mutant strains. However, it is difficult to reconcile this model with the observation that vancomycin does not alter the enzymatic activities of VanS_A_ in vitro [183], suggesting that more complex models may be required to explain glycopeptide sensing by VanS_A_.

### 3.5. Phylogenies of VanRS Proteins 

**Figure 6:**
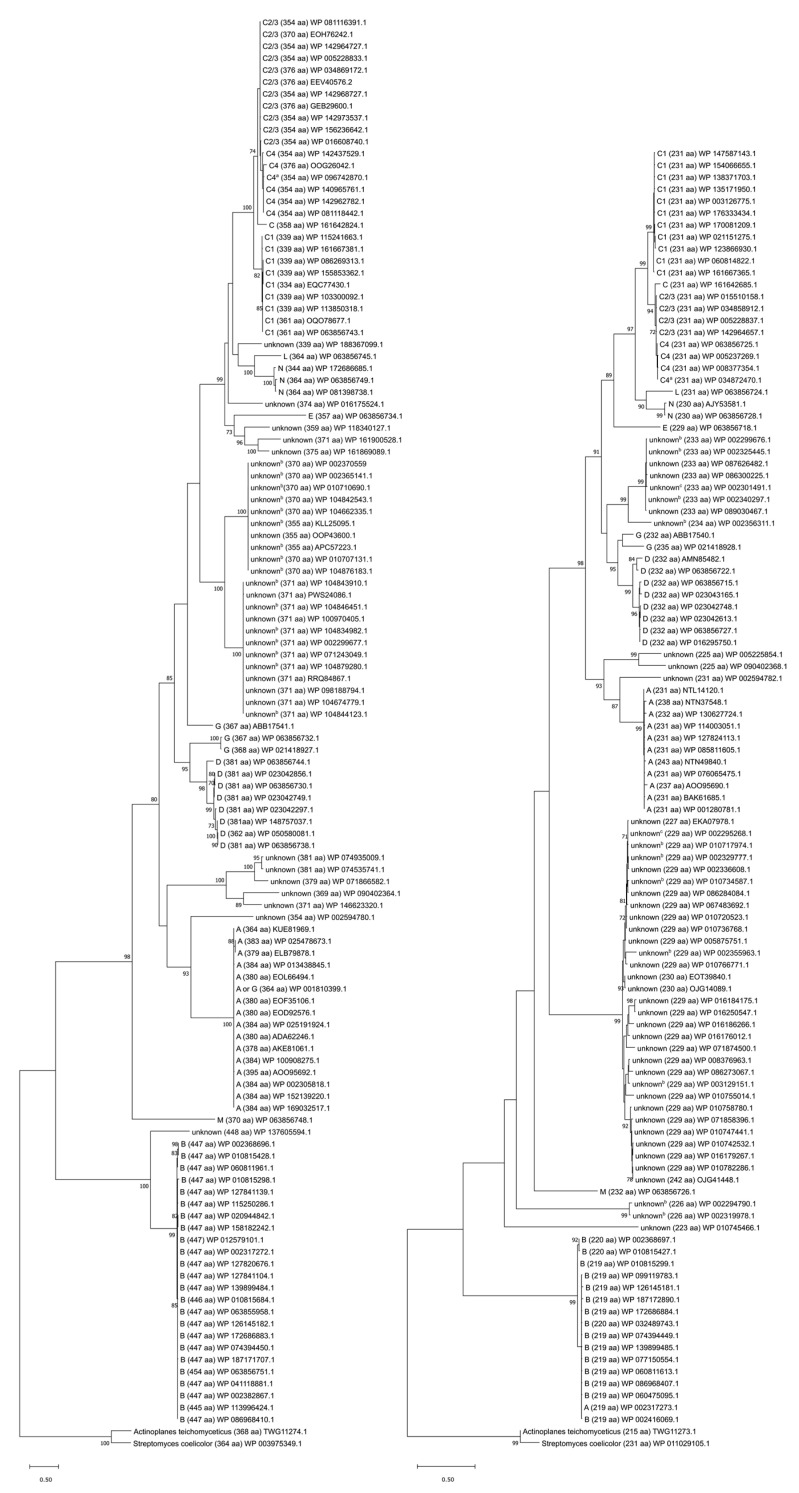
Evolutionary analysis of VanRS protein sequences. Trees are shown for VanS (**left**) and VanR (**right**), with VRE type, protein sequence length, and accession numbers for representative, nonredundant protein sequence entries being listed to the right of each branch. For the most part, C-type and -subtype proteins were not annotated as such, so they were subtyped based on nucleotide identity of *vanC* genes to type C1 *E. gallinarum* strain BM4174, type C2/3 *E. casseliflavus* strain ATCC25788, and type C4 *E. casseliflavus* strain F32 (Accessions: AF162694, L29638, and EU151752.1). Trees are rooted with sequences from *S. coelicolor* and *A. teichomyceticus*. The scale bar represents genetic distance equivalent to 0.5 substitution per site. Trees were constructed in MEGA X [91], using 120 and 109 MUSCLE-aligned sequences for VanS and VanR, respectively [89], and employing a maximum likelihood, LG+G model [90]. 200 iterations were used, with bootstrap values indicated at branch nodes. Bootstrap values < 70% are not shown. ^a^ Characterized by Watanabe et al. [108] as type VanC-4 despite higher nucleotide identity of the *vanC* gene to that of VanC-2/3-type *E. casseliflavus* strain ATCC25788. ^b^ VanRS proteins of unknown type belonging to Enterococcus strains for which vancomycin sensitivity was reported [73,76,77,78,244,245,246,247,248]. ^c^ VanRS proteins of unknown type belonging to Enterococcus strains for which vancomycin resistance was reported [78,244].

To obtain a comprehensive view of the relationships among the enterococcal orthologs of VanS and VanR, we constructed phylogenetic trees for both proteins (Figure 6). Nonredundant amino-acid sequences for VanR and VanS were collected by searching the NCBI Identical Protein Groups database (https://www.ncbi.nlm.nih.gov/ipg; accessed on 1 December 2020) for “vanS AND enterococcus” and “vanR AND enterococcus.” We also searched for VRE type-specific entries that might have been missed in the initial search. Results were filtered to exclude entries containing “partial” sequences. The VanS and VanR protein sequences from *S. coelicolor* were included in the final sequence analysis, as were those from the glycopeptide producer *A. teichomyceticus*, bringing the total sequence counts to 120 and 109 for VanS and VanR, respectively. *S. coelicolor* was included in the analysis because its VanS and VanR proteins have been extensively characterized, while *A. teichomyceticus* was included because it produces teicoplanin, and the operons conferring resistance to glycopeptide antibiotics are thought to have originated from such antibiotic-producing species [236]. The natural producer of vancomycin (*Amycolatopsis orientalis*) does contain a TCS that has been suggested to be involved with vancomycin resistance [237]; however, this is yet to be verified, and thus these genes were omitted from the analysis.

Conventionally, VRE are typed based on the gene sequence encoding their d-Ala-d-Lac/d-Ala-d-Ser ligase, and we followed this convention to assign VRE types for our VanS and VanR protein sequences. Although several VRE types have been divided into subtypes, we chose to subtype only C-type VRE, as the C subtypes are phenotypically distinct. Proteins were assigned to type C1 if they belonged to a strain with a vanC gene of 98–100% sequence identity to C1-type *E. gallinarum* strain BM4174, and 69–71% identity to C2/3 and C4 *E. casseliflavus* strains ATCC25788 and F32, respectively. C2/3-type proteins were classified as such if the *vanC* gene had 71% sequence identity to BM4174, 99–100% to ATCC25788, and 94% to F32. Proteins were assigned to type C4 if the *vanC* gene had 68–71% identity to BM4174, 94–96% to ATCC25788, and 96–98% to F32. The first of each type to be characterized was chosen as the reference strain [44,47,85].

This analysis contained proteins belonging to several vancomycin-susceptible enterococci [238,239,240,241,242,243,244,245,246], specifically *E. faecium*, *E. faecalis*, *E. mundtii*, *E. alcedinis*, *E. sacchoralyticus*, *E. asini*, *E. sp*. CU9D, *E. diestrammenae*, *E. malodoratus*, and *E. florum*. While these sequences were included because of their similarity to known VanRS proteins, they belong to strains that have not been explicitly identified as VRE [238,246], and which do not contain genes annotated as d-Ala-d-Lac/d-Ala-d-Ser ligases; hence it is currently unknown whether the corresponding gene products function as true VanRS proteins.

Although VRE are not typed based on their VanS and VanR sequences, it is unsurprising that in our phylogenetic analysis the VanS and VanR proteins cluster according to VRE type (Figure 6), suggesting that the regulatory proteins share common origins with the remainder of resistance operon. However, VanS and VanR do not appear to cluster on the basis of inducibility, which is the major phenotype associated with the regulatory proteins. For example, VRE types that generally express vancomycin resistance constitutively (C-, D-, and N-types) do not all cluster near one another, though the C- and N-type sequences appear to have diverged from one another relatively recently. Furthermore, sequences from VRE types in which resistance can be induced by both teicoplanin and vancomycin (A-, D-, and M-types) do not cluster either, suggesting that the regulatory proteins have arrived at their inducibility behavior by multiple avenues. 

The analysis highlights the high degree of similarity between the VanR and VanS proteins from *A. teichomyceticus* and *S. coelicolor*. Type B is the VRE type for which the VanR and VanS proteins are most similar to their counterparts in *A. teichomyceticus* and *S. coelicolor* (Figure 6). Consistent with this observation, the VanS proteins from type-B VRE and *S. coelicolor* exhibit functional similarities; despite sharing only 27% sequence identity, both VanS_B_ and VanS_Sc_ appear to interact directly with vancomycin (Section 3.3), and both respond to vancomycin, but not to teicoplanin [247]. However, this functional similarity does not appear to extend to *A. teichomyceticus*, since that organism is highly resistant to teicoplanin [248]. It is therefore difficult to infer detailed regulatory mechanisms from the phylogenetic relationships between different vancomycin-resistance regulators.

## 4. Conclusions

VanRS was established as the regulatory TCS of vancomycin resistance expression in 1992 [124]. Since then, studies of vancomycin resistance have made considerable progress in characterizing VanRS. However, because VRE pose a significant and growing threat to human health, a better understanding is required for how expression of the resistance phenotype is regulated. This requires addressing several key questions:

1. What is the mechanism of VanS activation for clinically relevant VanS orthologs? To date, while much progress has been made toward elucidating mechanisms for VanS_B_ and VanS_A_, definitive models still elude us; additionally, the heterogeneous nature of VRE suggests that additional mechanisms may prove relevant. Hence, there is a clear need for further biochemical and biophysical exploration of the activation mechanism(s).
2. What are the vancomycin-binding determinants for directly-activated VanS proteins such as VanS_B_? It now appears evident that VanS_B_ binds vancomycin via its periplasmic domain, leading to direct activation; however, the precise location must be mapped.
3. What are the structural consequences of activation for VanRS proteins? A recent structure of VanR_Sc_ is the first for any VanRS protein, but structural characterization lags for the VRE orthologs of VanR and VanS. Structures of these proteins will prove invaluable in any efforts to disrupt vancomycin sensing in VRE.

Disrupting the expression of vancomycin resistance is a potentially powerful new approach to restoring vancomycin susceptibility to VRE. Antibiotic adjuvants can be imagined that would abrogate VanS’s vancomycin-sensing activity or VanR’s DNA-binding activity; either should restore vancomycin susceptibility to VRE. An additional possibility is the development of novel glycopeptide antibiotics that retain vancomycin’s mechanism of action, but evade detection by VanS. Ultimately, however, any effort to modulate the expression of vancomycin resistance leads directly to the VanRS TCS.

## Figures and Tables

**Table 1:** Characteristics of vancomycin resistance in the nine types of VRE.

VRE Type	Terminal Dipeptide	Inducible	MIC Vancomycin (µg/mL)	MIC Teicoplanin (µg/mL)	Acquired	Transferable	Species	References
A	d-Ala-d-Lac	Yes	64 to >1000	16 to 512	Yes	Yes	*E. faecalis*, *E. faecium*	[15,16,30,36,38,39,68,69,70,71,72,73]
B	d-Ala-d-Lac	Yes	4 to 1024	≤0.5	Yes	Yes	*E. faecalis*, *E. faecium*	[33,43,68,73,74,75,76,77,78,79]
C	d-Ala-d-Ser	Yes/No	2 to 32	≤0.5 to 1	No	No	*E. gallinarum*, *E. casseliflavus*/*flavescens*	[32,33,34,46,56,80,81,82,83,84,85,86]
D	d-Ala-d-Lac	No	16 to 256	0.25 to 64	Yes	No	*E. faecalis*, *E. faecium*	[45,87,88,89,90,91,92]
E	d-Ala-d-Ser	Yes	16	0.5	Yes	No	*E. faecalis*	[48,49,93,94,95]
G	d-Ala-d-Ser	Yes	16	0.5	Yes	Yes	*E. faecalis*, *E. faecium*	[50,51,96,97,98]
L	d-Ala-d-Ser	Yes	8	N/A	Yes	No	*E. faecalis*	[99]
M	d-Ala-d-Lac	Yes	128 to 512	0.5 to >256	Yes	Yes	*E. faecium*	[52,100,101]
N	d-Ala-d-Ser	No	12 to 16	0.5	Yes	Yes	*E. faecium*	[53,102]

**Table 2:** SMART-predicted domains of the VRE VanS proteins [155].

		Location and Length							
VanS Ortholog	Protein Sequence Accession ^a^	TMH1 ^b^	TMH2	Periplasmic Domain	Linker Region	HAMP Domain	DHp Domain	Histidine Phospho-Acceptor (Residue Number)	CA Domain
A	WP_002305818.1 [41]	19–41 (23 aa)	78–97 (20 aa)	42–77 (26 aa)	98–153 (56 aa)		154–221 (68 aa)	164	266–376 (111 aa)
B	WP_002368696.1 [42]	7–29 (23 aa)	133–155 (23 aa)	30–132 (103 aa)	156–222 (67 aa)	157–208 (52 aa)	223–289 (67 aa)	233	334–445 (112 aa)
C1	WP_063856733.1 [44]	1–17 (17 aa)	37–56 (20 aa)	18–36 (23 aa)	57–114 (58 aa)		115–182 (68 aa)	125	227–337 (111 aa)
C2/3	WP_016608740.1 [47]	4–23 (20 aa)	36–58 (23 aa)	24–35 (12 aa)	59–114 (56 aa)		115–182 (68 aa)	125	227–337 (111 aa)
C4	ABX79412.1 (222)	4–23 (20 aa)	36–58 (23 aa)	24–35 (12 aa)	59–114 (56 aa)		115–182 (68 aa)	125	227–337 (111 aa)
D	WP_063856730.1 [45]	21–43 (23 aa)	76–98 (23 aa)	44–75 (32 aa)	99–155 (57 aa)		156–223 (68 aa)	166	268–379 (112 aa)
E	WP_063856734.1 [49]	13–35 (23 aa)	55–77 (23 aa)	36–54 (19 aa)	78–134 (57 aa)		135–205 (71 aa)	145	250–357 (108 aa)
G	WP_063856732.1 [50]	12–34 (23 aa)	70–90 (21 aa)	35–69 (35 aa)	91–144 (54 aa)		145–212 (68 aa)	155	257–366 (110 aa)
L	WP_063856745.1 [99]	17–39 (23 aa)	66–88 (23 aa)	40–65 (26 aa)	89–140 (52 aa)		141–208 (68 aa)	151	253–364 (112 aa)
M	WP_063856748.1 [52]	12–31 (20 aa)	57–79 (23 aa)	32–56 (35 aa)	80–140 (61 aa)	81–133 (53 aa)	141–208 (68 aa)	151	253–364 (112 aa)
N	WP_063856749.1 [53]	15–37 (23 aa)	61–83 (23 aa)	38–60 (23 aa)	84–140 (57 aa)		141–208 (68 aa)	151	253–364 (112 aa)

^a^ Representative VanS protein sequences were chosen because they belong to the first resistance gene cluster of each type to be characterized. For E-type, the accession listed is that of VanS_E_ from strain N00-410. There is no VanS_E_ protein sequence available from strain BM4405, the first E-type strain to have its resistance gene cluster characterized. ^b^ Numbers in each entry correspond to the range of residue numbers forming the relevant domain.
